# The effect of bio-electro-magnetic-energy-regulation therapy on sleep duration and sleep quality among elite players in Norwegian women’s football

**DOI:** 10.3389/fpsyg.2023.1230281

**Published:** 2023-08-08

**Authors:** Frode Moen, Svein Arne Pettersen, Kine Gjertsås, Marte Vatn, Martijn Ravenhorst, Atle Kvålsvoll, Kristian Hovde Liland, Ellen F. Mosleth

**Affiliations:** ^1^Department of Education and Lifelong Learning, Faculty of Social and Educational Sciences, Norwegian University of Science and Technology, Trondheim, Norway; ^2^School of Sport Sciences, Faculty of Health Sciences, UiT, The Arctic University of Norway, Tromsø, Norway; ^3^Department of Neuromedicine and Movement Science, Faculty of Medicine and Health Sciences, Norwegian University of Science and Technology, Trondheim, Norway; ^4^Department of Sociology and Political Science, Faculty of Social and Educational Sciences, Norwegian University of Science and Technology, Trondheim, Norway; ^5^The Norwegian Olympic Sport Center, Trondheim, Norway; ^6^Faculty of Science and Technology, Norwegian University of Life Sciences, Ås, Norway; ^7^Nofima AS - The Norwegian Institute of Food, Fisheries and Aquaculture Research, Ås, Norway

**Keywords:** sleep, elite football, recovery, physical load, BEMER therapy

## Abstract

The current study investigated if physical loads peak on game days and if Bio-Electro-Magnetic-Energy-Regulation (BEMER) therapy is affecting sleep duration and sleep quality on nights related to game nights among elite players in Norwegian women’s elite football. The sample included 21 female football players from an elite top series club with a mean age of ~24 years (± 2.8). Sleep was measured every day over a period of 273 consecutive days with a Somnofy sleep monitor based on ultra-wideband (IR-UWB) pulse radar and Doppler technology. The current study was conducted as a quasi-experiment, where each player was their own control based on a control period that lasted for 3 months, and an experimental period that lasted for 5 months. Accordantly, the time each player spent with BEMER therapy was used as a control variable. Multivariate analyses of variance using FFMANOVA and univariate ANOVA with False Discovery Rate adjusted *p*-values show that physical performance (total distance, distance per minute, sprint meters >22.5 kmh, accelerations and decelerations) significantly peak on game day compared with ordinary training days and days related to game days. The results also show that sleep quantity and quality are significantly reduced on game night, which indicate disturbed sleep caused by the peak in physical load. Most sleep variables significantly increased in the experiment period, where BEMER therapy was used, compared to the control period before the introduction of BEMER therapy. Further, the analyses show that players who spent BEMER therapy >440 h had the most positive effects on their sleep, and that these effects were significantly compared to the players who used BEMER therapy <440 h. The findings are discussed based on the function of sleep and the different sleep stages have on recovery.

## 1. Introduction

Elite women’s football (soccer) is an open loop sport that involves demanding physical actions as well as high mental loads (Nédélec et al., 2012; Jordet et al., 2020; Baptista et al., 2022). The physical demands in men’s and women’s elite football involve high occurrences of high-intensity actions, such as accelerations and decelerations, rapid change of direction, jumps, high-intensity runs and sprints, and direct contacts with opposing players (Datson, 2016; Marqués-Jiménez et al., 2017; UEFA, 2021; Winther et al., 2021). Further, the mental demands in football involve the players’ ability to perceive the constantly shifting environment on the field, quickly decide which action that is favorable in the play-counter play, and quickly execute the decisions made to try to outplay the opponent team (Williams, 2000). Thus, focused attention, shifting attention, the recognition of effective decisions stored in episodic memory (learned actions in similar situations), and making rapid decisions based on these perceptual-cognitive processes, are key mental processes in elite football. Such physical and mental efforts might lead to disturbances in the players’ physiology and neural processes (Nédélec et al., 2013).

Since elite women’s football has developed to exhibit more powerful and faster physical movements- (accelerations, decelerations, high-intensity runs, sprinting, jumps) and quicker and more rapid decisions from the football players, football training sessions must facilitate the development of such demands (Díaz-García et al., 2023). Taken together, the high physical-and mental loads in elite women’s football, especially during game-specific training sessions and football games, are demanding. The physical effort may lead to muscle fiber micro ruptures and muscular fatigue (Peake et al., 2017), and the excessive mental effort may lead to perceptual-cognitive overload (Jordet et al., 2020; Díaz-García et al., 2023). It is important to emphasize that there are substantial differences in high-intensity actions between playing positions in women’s elite football, with central defenders exhibiting lower relative distance and the fewest high-intensity activity bouts in distance and duration compared with all other outfield positions (Datson, 2016; Winther et al., 2021). Additionally, within-player sprint variability between official games can be more than 32% without being considered abnormal (Baptista et al., 2022). Nevertheless, the players train according to the various game demands in the different positions. Female football players are therefore exposed to a constant struggle to optimize the balance between the extensive load they are exposed to, and the recovery needed to keep fresh without reducing fitness (Fullagar et al., 2015; Moen et al., 2021).

Research shows that competition days in different sports affect subsequent sleep, since competitions are associated with reduced sleep quality and more sleep disturbances caused by the high physical loads in competitions (Fullagar et al., 2015; Knufinke et al., 2018; Biggins et al., 2020). Recent research has also observed increase in physical game-play performances of elite senior female players and that these physical and mental efforts peak significantly during football games, and that football games leads to acute fatigue that reduces physical performance ability over the following hours and days [Ispirlidis et al., 2008; Fédération Internationale de Football Association (FIFA), 2019; Lathlean et al., 2019; Winther et al., 2021]. Thus, to maintain and further develop the elite football players’ performance, they need optimal recovery to compensate for the physical and mental load (Bird, 2013; Smith et al., 2018). Studies show that sleep is disturbed on the nights close to game nights and game days in football are also found to reduce sleep quantity and quality (Fullagar et al., 2016; Roberts et al., 2019; Moen et al., 2021; Silva et al., 2022). Even though evening football games are found to have a negative effect on sleep (Sargent and Roach, 2016; Lastella et al., 2018; Nédélec et al., 2019), studies also claim that the game itself affect sleep more than the starting hour of the game (Lalor et al., 2018). Carriço et al. (2018) revealed a significant reduction of 65 min in sleep duration following female elite football games at both daytime and evening, compared to ordinary training days, where sleep duration averaged 6 h and 36 min. However, in general, there is a scarcity of data on female elite football players and few studies have investigated how sleep and the different sleep stages are affected by football games (Moen et al., 2021).

The strenuous demands of football games and game-relevant training sessions, encompassing physical and mental exertion, give rise to significant physical and mental disruptions, requiring a considerable amount of time for recovery (de Hoyo et al., 2016; Scott et al., 2020). The physiological disturbances, such as training and game induced muscle fiber micro ruptures and muscular fatigue, have a notable increase the first 24 h after a game, peak between 24 and 48 h, and then subside (Ascensão et al., 2008; Magalhães et al., 2010). The football players usually need from 72 to 96 h to restore metabolic homeostasis (Ispirlidis et al., 2008). The ability to gain optimal recovery in elite football is therefore of crucial importance to prevent the possibility of performance decrement (Andersson et al., 2008; Carling et al., 2012). Consequently, players who experience faster recovery will have a significant advantage by achieving quicker readiness for both training sessions and football games (Terrados et al., 2011).

The homeostasis and circadian rhythm are two internal biological processes that work together and provide the opportunity for important recovery processes to occur. The homeostasis process keeps track of the player’s need for recovery based on the physiological stress of different loads, and together with the circadian rhythm it regulates the players’ need for sleep. Sleep is the state where physiological processes necessary for recovery occurs and elite football players need to obtain both adequate quantity and quality of sleep to achieve optimal recovery (Venter, 2012; Nédélec et al., 2015; Moen et al., 2021). Sleep contributes to homeostatic control of energy conservation, and optimal functioning of the glymphatic, immune, metabolic, and endocrine systems (Bonnar et al., 2018). Physiological growth, repair, and neuromuscular performance are affected during sleep as well as brain homeostasis and neural plasticity, processing of emotional inputs and emotional regulation (Hrozanova, 2021). Thus, sleep is important for the restoration of the players’ physiology and their cognitive and behavioral performance, as well as their learning and memory capacity. Importantly, each sleep stage has a distinct complementary role in the overall human body recovery process (Vyazovskiy and Delogu, 2014). A recent study found that the night following football games were associated with reduced time in bed, total sleep time, time in all three sleep stages (light, deep, and REM), longer sleep onset latency and increased respiration rate in non-REM sleep (Moen et al., 2021). The study also found that players’ increase in perceived fatigue was associated with increased time in bed and deep sleep, and increased REM sleep was linked to a subsequently decreased in perceived fatigue (Moen et al., 2021). Thus, both the total amount of sleep football players gains and the distributed amount of time in the different sleep stages seem to be important in the recovery process to complete homeostasis. However, research claims that football players do not necessarily obtain enough sleep to become optimally recovered, especially after football games (Fullagar et al., 2015, 2016; Moen et al., 2021). Paradoxically, it is found that sleep among female elite football players is disturbed at the night after playing football games, and that this occurs at the same time as the need for recovery is increased (Moen et al., 2021). Thus, the disturbed sleep is claimed to be a result from the extraordinary loads associated with the football games.

Therefore, tools and methods that can monitor and influence functional recovery for elite football players are of high interest for players and coaching staff (Nédélec et al., 2012, 2013). Interestingly, research indicates that the use of Pulsed Electromagnetic Field (PEMF) therapy has the potential to influence human recovery through impacting tissue regeneration such as exercise-induced muscle damage (Basset, 1993; Nédélec et al., 2012, 2013; Ross et al., 2019). A recent study also claims that an increase in blood flow improves performance recovery between bouts of high-intensity exercise, and that PEMF might be an effective tool to achieve this (Borne et al., 2017). A physical vascular therapy based on PEMF that uses Bio-Electro-Magnetic-Energy-Regulation (BEMER) is found to have positive effects on several areas that are related to functional recovery (Bohn, 2013; Bohn et al., 2013). Therefore, BEMER therapy is considered to be a promising tool in therapeutic settings (Bohn, 2013). The use of PEMF therapy is mainly found to affect the blood flow in capillaries and the smallest blood vessels (microcirculation) in healthy muscles (Klopp, 2017). The heart needs help for the blood to flow through the capillaries (arterioles and venules) and capillaries are therefore equipped with a pump mechanism, where the tissue in the blood vessel walls contracts and extracts (relax) alternately to positively influence the blood flow (Intaglietta, 1990). When the body is exposed to stress this mechanism might be hampered (Pal et al., 1988). When the body is exposed to the weak pulsed electromagnetic fields produced by BEMER it is found to affect the rhythmical contraction–extraction mechanism in the blood vessel walls, and blood flow is increased (Bohn et al., 2013). This mechanism is defined as vasomotion. Due to an increased microcirculation PEMF and BEMER therapy is found to reduce pain (Benedetti et al., 2020), lower stress (Alzayed and Alsaadi, 2020), increased energy (Haase et al., 2011), and decreases inflammation and increases speed of regenerating homeostasis (Ross et al., 2019). Interestingly, BEMER therapy is also found to stimulate increase in quantity and quality of sleep (Bohn et al., 2013).

It is suggested that BEMER therapy might be beneficial in sports (BEMER Group, 2021), however scientific evidence is still missing (Lockie, 2021). Several studies claim that sleep is disturbed after football games and the nights close to game nights (Moen et al., 2021). Therefore, implementing a recovery tool that promotes the body to attain a normal sleep state following such demanding efforts would be effective in enhancing the overall recovery process. A recent study suggests that future studies should investigate the use of recovery strategies for improvements in sleep after football games, and that objective measurements should be used to detect workload, to investigate if the physical loads actually peak on game days compared to ordinary training days (Moen et al., 2021). The aim of the current study is to investigate objectively if physical loads peak on game days, and investigate whether BEMER therapy is affecting sleep duration and sleep quality among elite players in Norwegian women’s football? Since research has found that sleep is disturbed after games at the same time as the need for recovery has increased, the current study will focus on the days related to football games. The following hypothesis were developed:

*H1*: The physical loads in women’s elite football peak at game day.

*H2*: BEMER therapy is affecting the elite women’s football player’s sleep duration and sleep quality on game nights and the nights following game days.

## 2. Methods

### 2.1. Participants

Participants were recruited from a Norwegian female top level team in football. All players from the A team (25 players) together with their coaching staff were invited to an information meeting regarding the current research project. The aim of the research project was explained in detail, together with a description of responsibilities for the different tasks in the study, logistics during the project period, and the data collection process. The football players who decided to participate in the study signed a consent form approved by the local Regional Committee for Medical and Health Research Ethics (REC) in Central Norway (project ID 2017/2072/REK midt). Twenty-three football players returned signed forms and were enrolled in the study. However, one of the players decided to withdraw from the study after a month of data collection and one person used BEMER only 47 h for the whole experimental period and these two players were therefore omitted from the study. Thus, twenty-one football players (mean age 23.68 ± 2.8, range 20–29 years) participated in the current study. It was important to invite players from the same team to control the content of training sessions and games for the participants, and additional participants were therefore not available.

### 2.2. Instruments

The data collection lasted from the 1st of February 2022 until the 31st of October 2022 and covered both the pre-season and the complete official game season for the team. The relevant measurements for the current study are a measurement that detect objective sleep and a measurement that detect objective physical loads.

#### 2.2.1. Sleep

The Somnofy sleep monitor (version 0.7, VitalThings AS, Norway) is a novel, fully unobtrusive tool for sleep assessment, utilizing an impulse radio ultra-wideband (IR-UWB) pulse radar and Doppler technology. Somnofy is certified according to the Federal Communication Commission (FCC) and “Conformité Européene” (CE). The IR-UWB radar emits radio wave pulses in the electromagnetic spectrum, which can pass through soft materials (e.g., clothes or duvets), but are reflected by denser materials (e.g., a human body). As the pulses are reflected, they are returned and received by the IR-UWB radar again. Then, time-of-flight is used to analyze the time it takes to cover the distance between the radar and the object (the person), and then back to the radar. The movement of the sleeping person and their respiration rate are derived from the IR-UWB radar by utilizing the Doppler effect and Fast Fourier Transform, allowing the Somnofy to monitor the movement and respiration of the individual in bed with high precision. The raw movement and respiration data are processed by a sleep algorithm, which uses machine learning to calculate relevant sleep variables. Recently, a full validation of Somnofy against manually scored Polysomnography (PSG) found that Somnofy is an adequate measure of sleep and wake, as well as sleep stages, in a healthy adult population (Toften et al., 2020). Sleep studies in athletes, investigating the associations between sleep variables, sleep stages and physical and mental loads, have shown the Somnofy to be appropriate for use in athletic populations (Hrozanova et al., 2018, 2020; Moen et al., 2020). Table 1 shows the sleep variables that were obtained from the Somnofy sleep monitor in the current study.

**Table 1:** Sleep variables derived from the SOMNOFY^®^ sleep monitor.

Sleep variable	Unit	Description
Sleep onset (SON)	hh:mm	Time of day of sleep start (first onset of the night)
Sleep offset (SOF)	hh:mm	Time of day of wake-up (last wake-up of the night)
Time in bed (TIB)	h	The period between bedtime and get-up-time, including sleep and awake
Sleep onset latency (SOL)	h	The time it takes for the participant to fall asleep from when they intend to sleep
Total sleep time (TST)	h	Total sleep time from sleep onset to time at wake-up
Light sleep (LSL)	h/%^*^	Total amount of time in the light stages of sleep
Deep sleep (DSL)	h/%^*^	Total amount of time in deep sleep
Rapid eye movement sleep (REM)	h/%^*^	Total amount of time in REM sleep
Wake after sleep onset (WSO)	h	Times awake after sleep onset and before final awakening
Sleep efficiency (SEF)	%	The percentage of time from SON to SOF spent asleep
Respiration rate (RPR)	N	The number of respiratory ventilations in 1 min in non-REM sleep

^*^Percentage of sleep stage in relation to total sleep time.

#### 2.2.2. Physical load–GPS tracking

To quantify movement patterns and physical demands the football players were equipped with the FIFA-approved STATSports APEX (Statsports, Northern Ireland) system. During both training and games, each football player wore a GPS tracker on their upper back in a tight-fitted vest. To minimize inter-device errors, each player used the same GPS unit during the entire data collection period. After each training session and game, the recorded data from the units was retrieved and uploaded to the club’s laptop *via* the manufacturer software (STATSports Sonra 2.1.4). The complete dataset was exported to the researchers after the data collection period was finished. The raw data from each unit contains the id of the football player, in addition to time, latitude, longitude, Doppler-derived speed (m/s), heart rate (bpm), horizontal accuracy (Hacc), horizontal dilution of precision (Hdop), quality of signal, and instantaneous acceleration impulse, all captured at 10 Hz, while Micro-Electro-Mechanical-System data was captured at 100 Hz. The validity and levels of accuracy (bias <5%) of this tracking system have been previously presented (Beato et al., 2018). The variables that were collected for the purpose of the current study were the total sprint distance (meter) at higher speed than 22.5 km/h (SSM–High Intensity Sprint), total running/walking distance (TDI–Total Distance Meter), the number of accelerations higher than 2.26 m/s^−2^ (ACC–Accelerations), the number of decelerations higher than 2.26 m/s^−2^ (DEC–Decelerations), the amount of running/walking distance per minute (DPM), and the player’s maximal sprint speed (km/h^−1^) during training sessions and official games (PMS–Player’s Maximal Speed).

#### 2.2.3. BEMER therapy device

BEMER stands for Bio-Electro-Magnetic-Energy-Regulation and the device uses a pulsed electromagnetic field (PEMF) to deliver a patented bio-rhythmically defined therapeutic signal. The BEMER Essential-Set was delivered to each of the football players in the current study, and the set contains the BEMER Box Professional control unit, where the football players administrate the different therapy programs, the BEMER Body applicator for full-body application, and the BEMER Pad applicator for local body application. The BEMER Box Professional control unit registers the time therapy programs were used by the players. At the end of the study, each football player’s BEMER Box Professional control unit was analyzed to register the total amount of hours each football player used the BEMER therapy (BMT–BEMER Time).

### 2.3. Procedure

Once all participating football players returned the signed consent forms, the necessary equipment for sleep monitoring was delivered, along with instructions for correct use. The football players were instructed on the correct placement of the sleep monitor and the importance of correct settings for optimal functionality. The data collection entailed day-to-day monitoring of the football players’ sleep detected by the Somnofy sleep monitoring device and physical loads collected by the GPS STATSports APEX tracking system. The researchers had access to a real-time overview of participants’ compliance with the study and monitored the progress closely throughout the whole data collection to address and solve any technical issues in relation to the sleep monitoring systems and GPS STATSports APEX tracking system.

**Figure 1:**
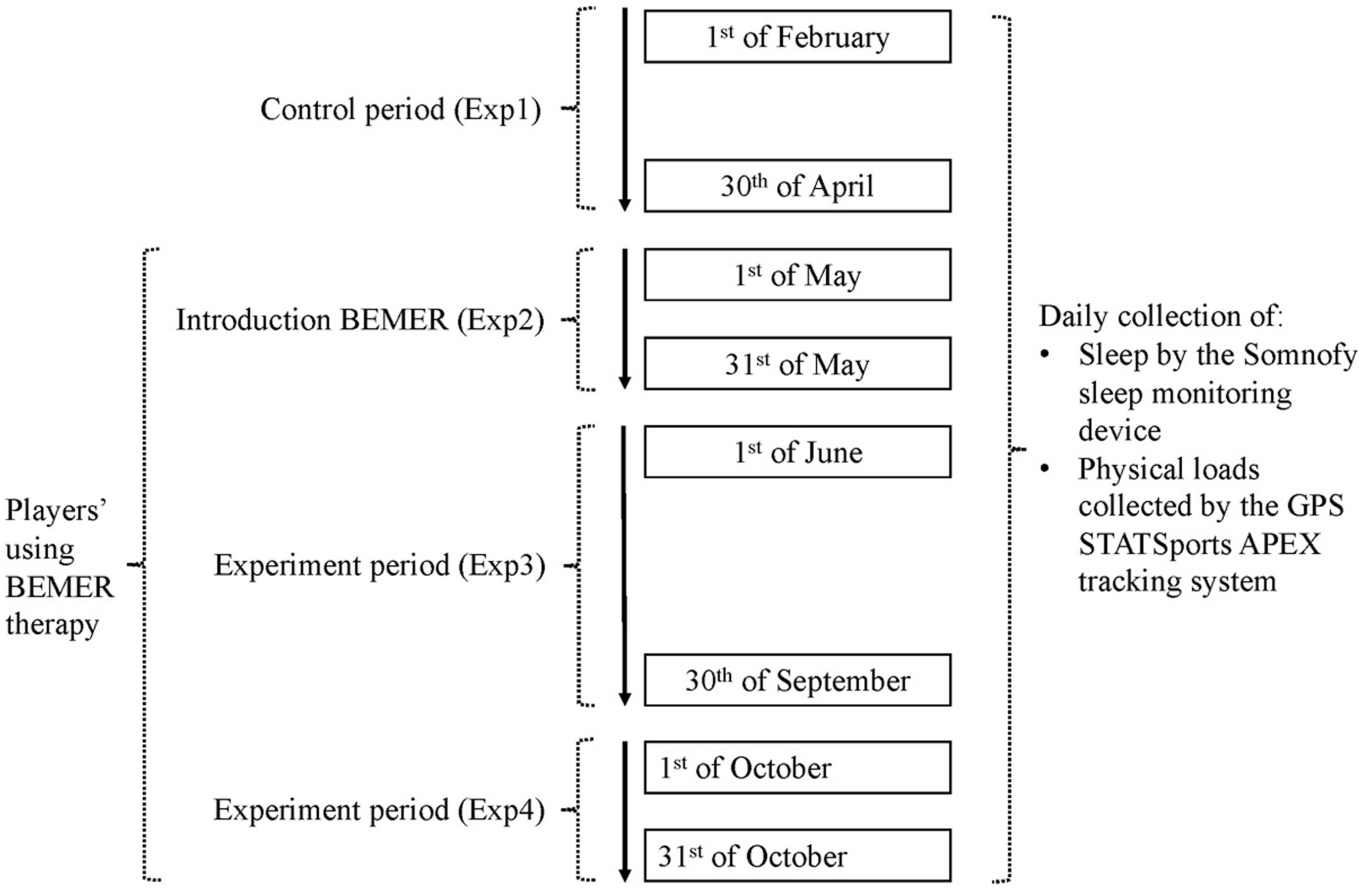
The different periods of data collection during the study.

The 273 data collection days of the current study were divided into four periods: Experimental period 1 (Exp1): the control period before the introduction of BEMER therapy lasted from the 1st of February until the 30th of April (89 days), Experimental period 2 (Exp2): introduction period for BEMER therapy from the 1st of May until the 31st of May (31 days), Experimental period 3 from the 1st of June until the 30th of September (Exp3): a period where the players were familiarized with using BEMER therapy under a period with a normal training week and games in the week ends (122 days), and thereafter Experimental period 4 from the 1st of October until the 31st of October (Exp4): a period using BEMER therapy in an extraordinal hard congested fixture period due to qualification to Champions League, play offs in the Norwegian national cup and official games in the series (31 days). Figure 1 shows the different periods in the current study.

#### 2.3.1. The BEMER protocol

The BEMER set was delivered to each football player after the 3 months control period (Exp1) was over (1^st^ of May). The football players were instructed to use the BEMER base program 8 min in the morning and 8 min in the evening every day (BEMER Group, 2020). The base program has 10 different intensities, and the football players were instructed to start with intensity 1 the first week and increase by one on the intensity scale for each week. At intensity 6 the players were instructed to go back to intensity 3 and increase for each week until they reached intensity 6. This base protocol was followed throughout the intervention period. The players were also instructed to use the BEMER therapy program after high intensity training sessions and football games. The BEMER therapy program has 3 levels, where level 1 lasts for 12 min, level 2 for 16 min and level 3 for 20 min. The first two weeks the players were instructed to use BEMER therapy program level 2, and the next weeks level 3. After 8 weeks the players were instructed to use the BEMER Special mode program during sleep. The players placed the BEMER Body applicator for full-body application mattress under their sleeping mattress, and the BEMER Special mode program allows the football players to set their weakening time on the BEMER Box Professional control unit, and the BEMER therapy will start 2 h before the players wake up. They were instructed to use this program once a week after 8 weeks with the base program, and increase with one session the second week, and use it for up to three times each week throughout the intervention period.

### 2.4. Statistical analyses

Initially, IBM SPSS (version 27.0) was used to conduct demographic and descriptive statistical analyses, which are presented as mean ± standard deviation (S.D.). Extreme outliers of sleep data, defined as epoc counts of sleep sessions that were below 3 h (1,000 s) and sleep sessions where time in no presence are higher than 3,000 s were deleted. Also, to avoid that movements in the room were detected as sleep data, sleep sessions between 12.00 after midnight and 20.00 before midnight were deleted.

**Figure 2:**
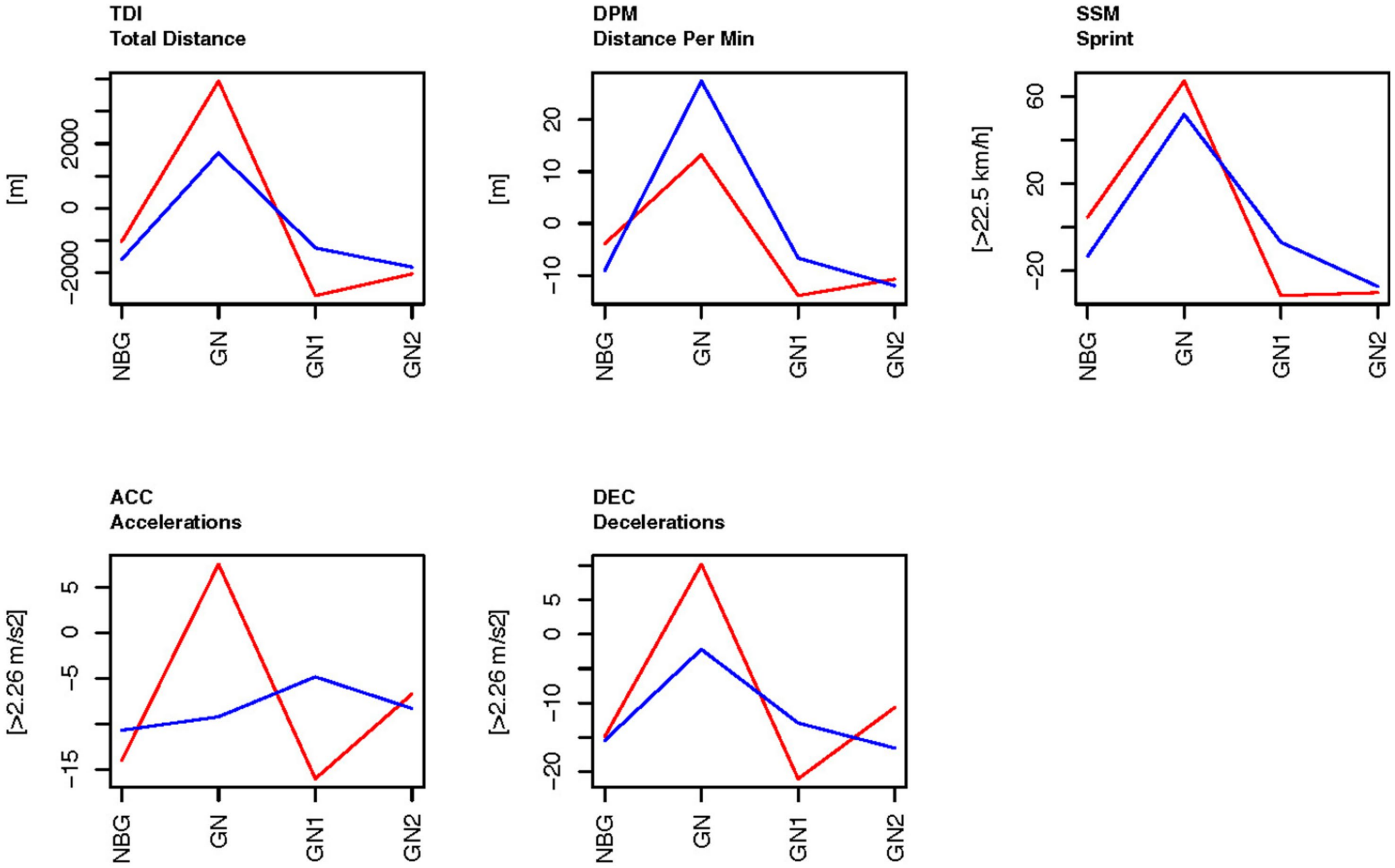
The physical load GPS-tracking on the days related to football games during the control period Exp1, before BEMER (red), and experimental period Exp3, the BEMER test period (blue). The data display each type of nights as a difference to the training nights (TN) for each person within the same experimental period. The baseline (0 on the *y*-axis) is means of the ordinary training days that were not close to a game (TN), as calculated for each person within each experimental period. TDI, total distance; DPM, distance per minute; SSM, sprint distance; ACC, Accelerations; DEC, Decelerations; NBG, night before game; GN, game night; GN1, night after GN; and GN2, night after GN1.

**Figure 3:**
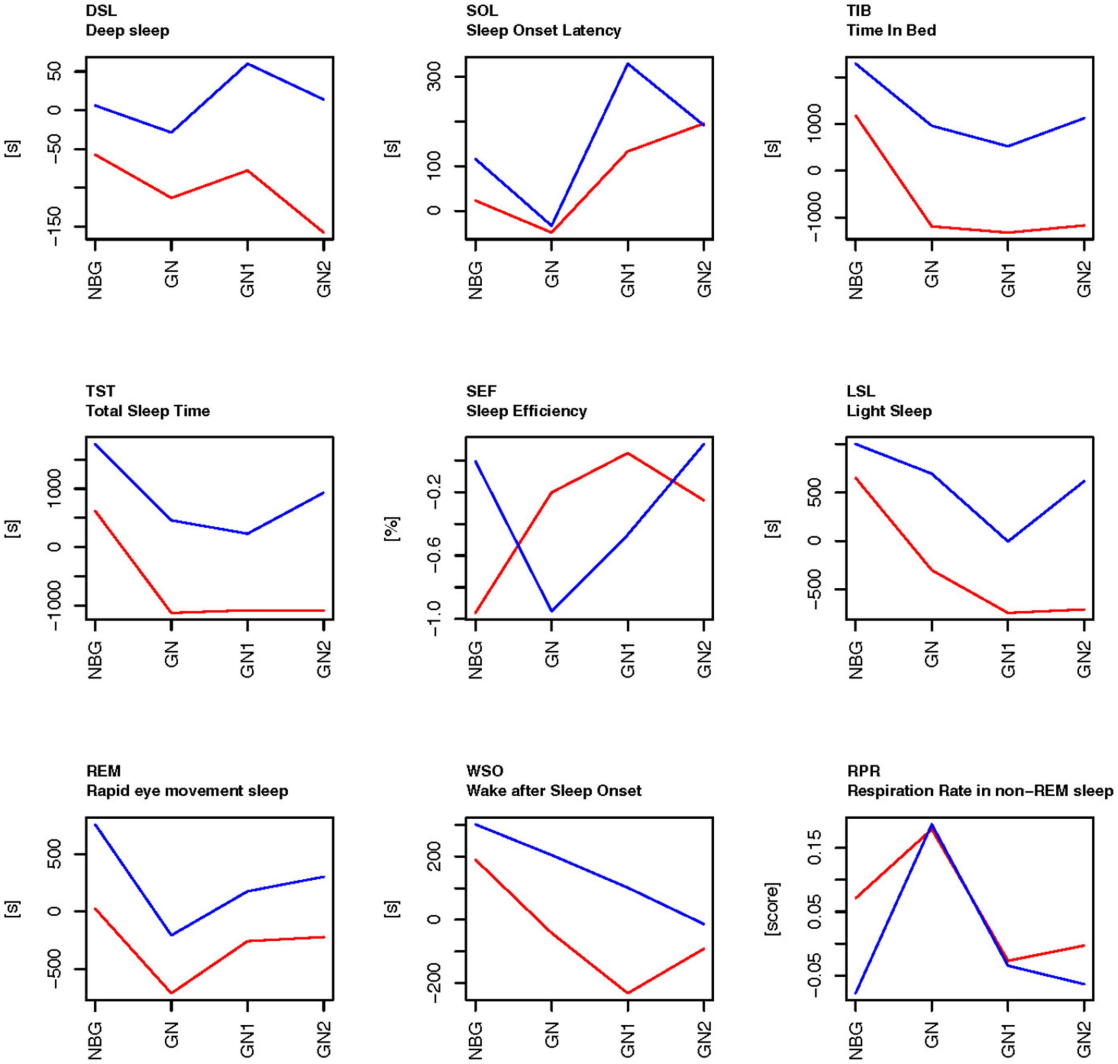
The sleep data on the days related to football games during the control period (Exp1-red) and experimental period (Exp3-blue), compared to the baseline scores on general training nights. The baseline (0 on the *y*-axis) is means of ordinary training days that were not close to a game (TN), as calculated for each person within each experimental period. NBG, night before game; GN, game night; GN1, night after GN; and GN2, night after GN1.

Within each experimental period (Exp1, Exp2, Exp3 and Exp4) the days/nights were classified as different types of days/nights: days/nights that were not close to a game and were ordinary training days where classified as training day/night (TN), night before game (NBG), game day/night (GN), game day/night plus 1 (GN1) or game day/night plus 2 (GN2) (Table 2 and Figures 2, 3). Each night is related to the day the need for sleep is developed. Thus, the day the football game is performed equals GN.

**Table 2:** Classification of days related to the players’ participation in football games.

TN	Nights after ordinary training sessions and not close to a football game: Training Nights
NBG	The night before a football game
GN	The night following the football game: Game night
GN1	The night 1day following game night: Game night +1
GN2	The night 2 days following the game night: Game night +2

On some rare occasions there were two successive games and two games with only one day in between, which resulted in a combination of the night classes presented in Table 2. These rare cases were omitted from the data as they were not balanced between the experimental periods. The different players used BEMER therapy in various degree. The real time they used BEMER therapy was collected from the BEMER devices after data collection termination and incorporated in the statistical analysis by dividing the individuals into two groups: group 1 (B1) used BEMER more than 150 h and less than 440 h during the experimental period, and group 2 (B2) used BEMER more than 440 h. The football players were then classified by a variable B-level, based on if they had used BEMER below 440 h (*N* = 6) or more than 440 h (*N* = 15).

For each group of variables (physical load data and sleep data), days without observations were omitted. This resulted in 1928 observations for physical data and 3,883 observations of sleep data. For each football player within each experimental period, the means of general training days was used as baselines, and the observed values of nights close to football games (NBG, GN, GN1 and GN2) were considered as a difference to the players’ mean values of TN. Experimental period 2 (Exp2) was the introduction period of BEMER therapy and was omitted from these analyzes. Furthermore, because of significant data loss in Exp4, occasions of successive games with or without only one day between and less days in Exp4 compared with Exp1 and Exp3, data from Exp4 was omitted from the further analyzes.

These data were analyzed as three-ways factorial design by univariate analysis of variance ANOVA with False Discovery Rate adjusted *p*-values (Moen et al., 2005) and by multivariate analysis of variance using FFMANOVA (Langsrud, 2003, 2005). The three design variables in the analyses of variance were:

1. Type: NBG, GN GN1, or GN2
2. Exp: Control period Exp1 or experimental period Exp3
3. B-level: 150–440 h or above 440 h total BEMER time

Each of these three factors and all two-ways interaction between them were used as design variables in the analysis of variance. The analysis was performed in R version 4.2.1 using the ffmanova R package^1^ (Langsrud et al., 2007). FFMANOVA gives a validation of all parameters simultaneously by performing Principal Component Analysis (PCA), where the first and most important PC’s are validated toward the PC’s reflecting noise in the data. Separate analyses were performed for physical activity and for sleep observations using the design variables Type, Exp and B-level observed as a difference to means of the training nights within each player in Exp1.

## 3. Results

The current study had a total potential of collecting 6,006 data points including sleep and physical performance data using GPS tracking over the 273 days. For the objective sleep data, 4,746 or 79%, of the potential 6,006 data points were collected and available for analyses. Missing data was a result of occasional disengagement of the football players with the sleep monitoring systems, technical issues with connecting the Somnofy units to Wi-Fi (especially at some hotels), travels where the football players forgot their sleep monitoring devices at home, and one of the football players was transferred to another club in July. Of the 6,006 potential data points of physical performance data, 2,497 or 41,6%, were collected. Data was lost due to training sessions where the GPS tracking vest was not appropriate to use (example strength training), and to the players’ occasional forgetfulness. Overall, a total of 406 observations from training and official football games were investigated for the 273 consecutive days, there were 339 occurrences where the football players played full time games (45 min x 2), 42 played one half or more (+ 45 min) and 80 played less than 30 min. Descriptive statistics (mean ± STD) of the studied sleep variables during the entire period of data collection including all football players, and at game nights are shown in Table 3.

**Table 3:** Descriptive statistics for the objective sleep patterns, based on the total period of 4,746 nights of data in 21 females football players, and the night after completing full football games (252 nights), respectively.

Sleep variable	Mean (± STD)	Mean (± STD) game nights
SON	24:00 (± 00:58)	00:10 (± 00:59)
SOF	08:37 (± 01:06)	08:44 (± 01:05)
TIB	09:33 (± 01:21)	09:29 (± 01:20)
SOL	00:42 (± 00:30)	00:39 (± 00:28)
TST	08:02 (±01:11)	07:58 (± 01:14)
LSL	04:46 (59%) (±00:52)	04:53 (± 00:57)
DSL	01:19 (17%) (±00:22)	01:19 (± 00:23)
REM	01:57 (24%) (±00:33)	01:45 (± 00:31)
WSO	00:34 (±00:32)	00:34 (± 00:31)
SEF	84.4 (±7.7)	84.1 (± 7.9)
RPR	15.4 (±1.9)	15.9 (± 2.3)

STD, standard deviation; SON, sleep onset; SOF, sleep offset; TIB, time in bed; SOL, sleep onset latency; TST, total sleep time; LSL, light sleep; DSL, deep sleep; REM, rapid eye movement; WSO, wake after sleep onset; SEF, sleep efficiency; RPR, non-rapid eye movement respiration per minute.

For the physical load data there were 389 datapoints in the control period Exp1 and 468 datapoints in the experimental period Exp3. For the sleep data there were 674 datapoints within control period Exp1 and 797 datapoints for the experimental period Exp3.

The studied physical load variables collected by the GPS STATSports APEX tracking system showed that the mean locomotor distance of the players during an average training session was 4,315 meters (±1863), they sprinted (speed above 22.5 km/h) a mean distance of 26 meters (±47), and their mean registered maximal speed was 23.3 km/h (± 3.7). During football games the mean distance for the football players that participated in the game was 9,313 meters (±4,743), they sprinted a mean distance of 127 meters (±107), and their mean registered maximal speed was 26.8 km/h (± 2.2). The football players’ mean of BEMER therapy use was 595.7 h (±252.9) during the experiment periods Exp2, Exp3 and Exp4.

For all physical variables there were significant main effects of type of day/night (Type), significant main effects of experimental period (Exp), and significant interaction between these factors (Table 4). Among the physical variables, distance per minute (DPM) is the only variable that is not influenced by the time the football players actually participated in the games or in the training sessions, but the relative locomotor distance of the players during the time they participated in the games or training sessions. Figure 2 shows that during a football game (GN), the DPM was higher than during normal training days (the baseline), both in the control period (Exp1) and in the experimental period (Exp3) (reflected by positive values for DPM at GN in Figure 2). Compared with the days before and after the game (NBG, GN1 and GN in Figure 2) there was a peak at the game day (GN) both for the experimental period (Exp3) and for the control period (Exp1), but a higher value for the experimental period (Exp3). For the days close to a game, the distance per minute were shorter than the means during normal training days (the baseline), which resulted in negative values in Figure 2. All other physical variables, except acceleration in the experiment period (Exp3), also peaked at the game day. At GN these variables were higher in the control period (Exp1) than in the experimental period (Exp3). However, these values were not controlled to be the relative locomotor effort players participated in the games or in training sessions.

**Table 4:** Multivariate and Univariate ANOVA using FFMANOVA for validation of changes in Physical load–GPS tracking variables on days close to football games (Type), the experimental periods (Exp), and the BEMER group (B-level) based on time spent on usage (across all variables and within each variable).

MANOVA across all variables			ANOVA within each variable				
Variable	DF	*p*-value	TDI	DPM	SSM	ACC	DEC
Type	1	**0.000**	**0.000**	**0.000**	**0.000**	**0.000**	**0.000**
Exp	1	**0.000**	**0.001**	**0.000**	**0.070**	**0.098**	**0.018**
B-level	3	**0.046**	0.186	0.831	0.154	0.324	0.300
Exp*Type	1	**0.000**	**0.000**	**0.000**	**0.011**	**0.000**	**0.000**
B-level*Exp	3	0.900	0.971	0.971	0.971	0.971	0.461
B-level*Type	3	**0.065**	**0.003**	0.747	0.138	0.138	**0.004**
Residuals	844						

DF, degrees of freedom; TDI, total distance; DPM, distance per minute; SSM, sprint distance; ACC, Accelerations; DEC, Decelerations; Type, days that was not close to a game (TN); day before game (NBG), gameday (GN), gameday + 1 (GN1) or gameday + 2 (GN2), B-level = (BEMER level) is on two levels: (1) < 440 h (2) > 440 h. Significant differences are in bold.

For the sleep variables there were significant main effects of type of days/nights (Type), and significant main effects of experimental period (Exp) for most sleep variables (Table 5). There were no significant interaction effects between these two factors. The sleep variables time in bed (TIB), total sleep time (TST) and light sleep (LSL) were all higher in the experimental period (Exp3) than in the control period (Exp1) at all types of days/nights: the game nights and the nights close to a game (Figure 3). In the control period Exp1, deep sleep (DSL), time in bed (TIB), total sleep time (TST), light sleep (LSL) and REM-sleep (REM) were below the baseline on game nights (GN) and the two nights following game nights (GN1 and GN2). There were also significant effects of type of days/nights with a drop from the night before a game (NBG) to the game nights (GN), both in the experimental period (Exp3) and in the control period (Exp1). The time in deep sleep (DSL) also showed a tendency of higher values in the experimental period (Exp3) than for the control period (Exp1) but did not reach statistical significance (Table 5).

**Table 5:** Multivariate and Univariate ANOVA using FFMANOVA for validation of changes in sleep variables on nights close to football games (Type), the experimental periods (Exp), and the BEMER group (B-level) based on time spent on usage (across all variables and within each variable).

MANOVA across all variables			ANOVA within each variable								
Variable	DF	*p*-value	SOL	TIB	TST	SEF	LSL	REM	DSL	WSO	RPR
Type	1	**0.000**	0.169	**0.000**	**0.000**	0.718	**0.000**	**0.000**	0.719	0.321	**0.000**
Exp	1	**0.000**	0.627	0.**000**	0.**000**	0.633	**0.000**	**0.000**	**0.090**	0.199	0.403
B-level	3	0.213	0.805	0.310	0.805	0.310	0.758	0.310	0.310	0.299	0.758
Exp*Type	1	0.714	0.915	0.391	0.527	0.527	0.379	0.883	0.915	0.883	0.527
B-level*Exp	3	**0.000**	**0.003**	0.597	**0.001**	**0.000**	**0.011**	**0.026**	**0.079**	**0.003**	0.373
B-level*Type	3	0.659	0.840	0.840	0.840	0.881	0.840	0.840	0.840	0.816	0.840
Residuals	1.457										

DF, degrees of freedom; SOL, sleep onset latency; TIB, time in bed; TST, total sleep time; SEF, sleep efficiency; LSL, light sleep; REM, rapid eye movement; DSL, deep sleep; WSO, wake after sleep onset; RPR, non-rapid eye movement respiration per minute; Type, nights that was not close to a game (TN), night before game (NBG), game night (GN), game night + 1 (GN1) or game night + 2 (GN2), B-level = (BEMER level) is on two levels: (1) < 440 h (2) > 440 h. Significant differences are in bold.

The amount of REM sleep was significantly higher in the experimental period (Exp3) than the control period (Exp1), and significantly higher on the night before game compared to their baseline scores within the experimental periods, whereas the amount of REM sleep droped to their baseline level on game night (GN) and increased on GN1 and GN2. In experimental period (Exp3), but not in the control period (Exp1), REM was above the baseline the day after the game, and the day thereafter. The result was similar in the control period (Exp1), but then the REM sleep levels are lower than their baseline scores. Wake after sleep onset (WSO) was above baseline the night before game and has a linear trend toward baseline level on GN2 in the experimental period (Exp3), whereas it was below baseline levels in the control period (Exp1) on GN and the two following nights. The respiration rate in non-REM sleep increased significantly on GN compared to the players’ baselines scores in both the control period and the experimental period.

**Figure 4:**
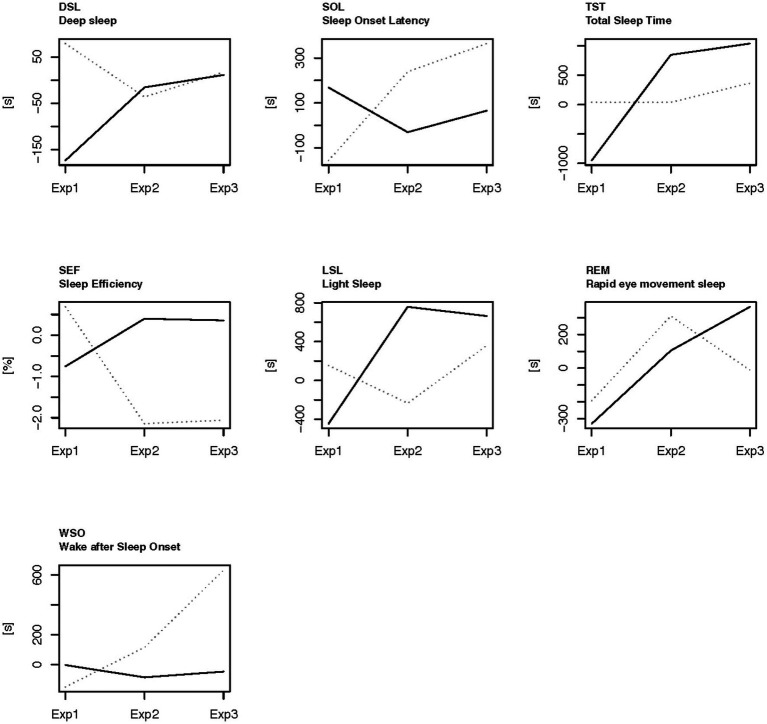
The sleep data detected in the different experimental periods for the players who used BEMER more than 440 h (marked line) and the players who used BEMER less than 440 h (dotted line). The baseline (0 on the *y*-axis) is means of the days of ordinary training days that were not close to a game (TN), as calculated for each person within each experimental period. Exp1, the control period; Exp2, introduction period for BEMER; Exp3, familiarized with the use of BEMER and using the BEMER; Marked line, players who used BEMER more than 440 h; Dotted line, players who used BEMER therapy less than 440 h.

There are interacting effects between the BEMER therapy group variable (B-level: the amount of time the players used BEMER therapy) and the scores over the experimental period for most of the sleep variables (Table 5). For sleep variables with significant interaction effects between B-level and Exp, Figure 4 shows the results of means for the players that used BEMER >440 h and those that used BEMER <440 h.

Figure 4 shows that the players who had the most extensive exposure to BEMER therapy (>440 h) had significantly lower sleep onset latency (SOL) than the players who had fewer hours of BEMER therapy (<440 h), and that the value decreased from Exp1 to Exp2 and Exp3. In experimental period Exp3, the total sleep time (TST), sleep efficiency (SEF), and the amount of REM sleep are all significantly higher than in the group of players who spent less time on BEMER therapy. Wake after sleep onset (WSO) time was significantly lower for the group who spent a higher amount of time on BEMER therapy. The players who had the highest amount of time with BEMER therapy increased their deep sleep (DSL) significantly from Exp1 to Exp2 and Exp3.

**Figure 5:**
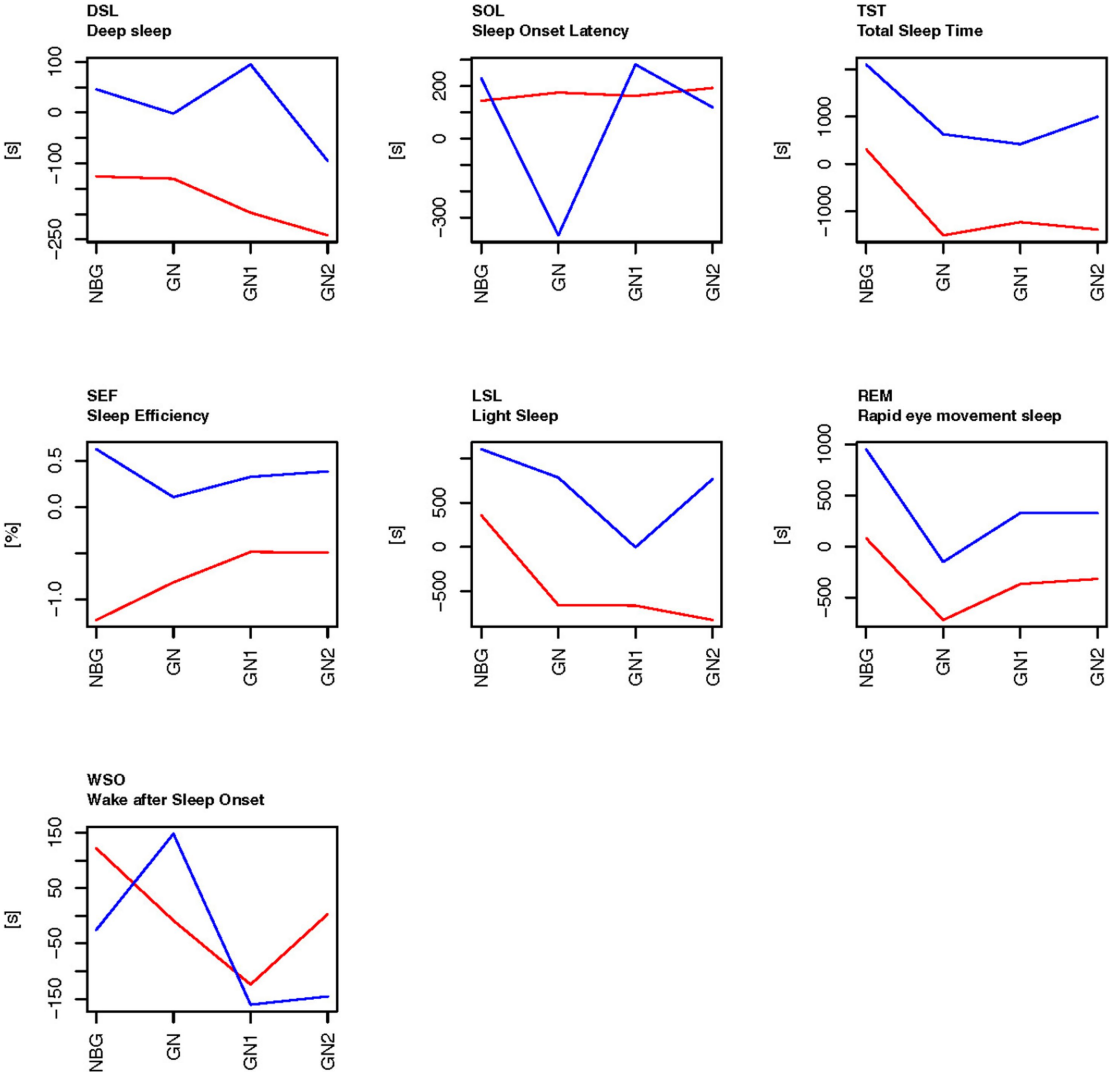
The sleep data on the days related to football games during the control period (Exp1-red) and experimental period (Exp3-blue), compared to the baseline scores on general training nights for the players who used BEMER therapy more than 440 h. The baseline (0 on the *y*-axis) is means of the days of ordinary training days that were not close to a game (TN), as calculated for each person within each experimental period. NBG, night before game; GN, game night; GN1, night after GN; GN2, night after GN1.

Figure 5 displays the results of the players that used BEMER >440 h (B2) on sleep variables prior to-and post football games in the control-and experimental period.

Figure 5 also shows that the pattern for the sleep variables TST, LSL, REM, and DSL share the same pattern as in Figure 3, where both groups are included in the analyses, whereas the sleep variable sleep onset latency (SOL) was significantly lower on game night in the experimental period (Exp3) for the players who used BEMER therapy more than 440 h than for those who used BEMER therapy less, and sleep efficiency (SEF) was above or close to the baseline levels on nights close to game nights in the experimental period (Exp3), but below the base line in the control period (Exp1).

## 4. Discussion

The aim of the current study was to investigate objectively if physical loads peak on game days, and whether Bio-Electro-Magnetic-Energy-Regulation (BEMER) therapy is affecting sleep duration and sleep quality among elite players in Norwegian women’s elite football. To the authors knowledge there are currently no studies that document possible effects of BEMER therapy on sleep in elite sports. The first hypothesis of the current study predicted that the physical demands in women’s elite football peak at football games. The prediction was confirmed whereas all collected physical variables significantly increased on game days compared to general training days. The second hypothesis predicted that BEMER therapy affected the elite women’s football player’s sleep duration and sleep quality on game nights and the nights following game days. The second hypothesis was also confirmed whereas the football players’ sleep duration and sleep quality significantly increased in the experiment period compared to the control period on game nights and the nights following game days.

### 4.1. Physical loads peak on game days in women’s football

As predicted in the hypothesis the results in the current study show that the physical loads tracked by the GPS tracking system (STATSports APEX) were significantly higher on game days compared to the statistical means of general training days, the day before game, the day after the game and two days after the game. Total running/walking distance, distance per minute, sprint meters above 22.5 km/h, and the number of decelerations peak on game days in both the control-and the experimental period. The number of accelerations peak on game days in the control period, but not in the experimental period. Accordingly, research claims that the mental loads on match days are significantly higher than normal training days, since both the cognitive-perceptual demands rises, as well as the emotional arousals associated with football games (Ispirlidis et al., 2008; Winther et al., 2021). Thus, both the physical-and mental loads peak on game days compared to all other training days, and thus the need for recovery becomes more important. The significant increase in respiration rate in non-REM sleep on game night compared to their baselines scores in both the control period and the experimental period, is also associated with stress from increased loads (Moen et al., 2021). However, the current study shows that both sleep quantity (total sleep time) and sleep quality (the time distributed in the different sleep stages and time awake after sleep onset) are reduced on game days, especially in the control period. This result is in line with earlier research that claims that sleep is disturbed on game nights because of a significantly peak in physical and mental loads (Fullagar et al., 2016; Lalor et al., 2018; Roberts et al., 2019; Moen et al., 2021). Improved sleep quantity and quality on game nights, and the nights following game nights, will therefore reflect a potential positive environment for more comprehensive recovery after such a significant increase in loads.

### 4.2. Improved recovery equals changes in sleep quantity and quality

The results from the current study show that total sleep time has significantly increased after the introduction of BEMER therapy compared to the control period on game nights (Table 5 and Figure 3). Time in bed has also increased significantly, and the significant increase in time in bed and total sleep time also applies to the nights before games, game nights, game nights +1 and + 2. Interestingly, total sleep time is significantly lower on game nights and game nights +1 and 2 than the players’ normal sleep status (Figure 3) in the control period, and above the players’ normal sleep status after the introduction of BEMER therapy. The decreased total sleep time that is detected in the control period on game nights can be explained by the physical and mental disturbances caused by the increased loads associated with the football games (Lalor et al., 2018; Moen et al., 2021). The significant increase in respiration in non-REM sleep on game nights strengthen this explanation, since raised respiration often is caused by an increase in physiological or psychological stress (Figure 3). The increased total sleep time after the introduction of BEMER therapy indicate that the players’ sleep is not interrupted in the same degree in the experimental period. Thus, the increased total sleep time is associated with the raised need for recovery caused by the peak in load associated with the football games, and that the players achieve their needs for recovery after games to a larger degree after the introduction of BEMER therapy. However, the patterns of the players’ sleep associated to football games (TIB, TST, LSL, REM, DSL) show that all sleep variables raise either the night after the game (game night +1), or the following night (game night +2), which indicate that the players’ still need to catch up their sleep to be in total balance after the loads associated with the game (Ispirlidis et al., 2008). Research claims that players need about 3 to 4 nights to restore metabolic homeostasis (Ispirlidis et al., 2008). The results in the current study also show that the players who used BEMER therapy more than 440 h significantly improved their total sleep in the experimental periods (Figure 4), and significantly more than the players who used BEMER therapy less (Table 5). Interestingly, their time spent in bed was not significantly different. Thus, the findings from the present study provides evidence to support the assertion that the introduction of BEMER therapy leads to a significant improvement in sleep quantity.

#### 4.2.1. Sleep quality

The attributes that often are used to define sleep quality are sleep efficiency, sleep onset latency, total sleep time, and wake after sleep onset (Nelson et al., 2022). Importantly, sleep quality must be related to the players’ acquired need for recovery, and sleep quality is also claimed to be impaired when there is reduction in deep sleep and REM sleep, in combination with increased time in light sleep (Berry, 2012). The results in the current study show that total sleep time, light sleep, REM sleep, and deep sleep are significantly reduced on game nights in the control period, and that all these variables are significantly higher after the introduction of BEMER therapy on game nights and all nights related to game nights. Total sleep time naturally affects the time spent in the different sleep stages, whereas longer total sleep time necessarily associates with longer durations in sleep stages, and longer total sleep time equals increased recovery. Each of the sleep stages fulfills a distinct role to the overall function of sleep, and at the same time the sleep stages have complimentary roles (Vyazovskiy and Delogu, 2014). However, sleep stage distribution in elite athletes is not yet fully understood (Moen et al., 2021).

In light sleep the brain waves, respiration and heart rate slows down, and the muscles become more relaxed compared to wakefulness (Singh and Stephen, 2022). These slow down activities help the physical restoration of the players. The development of motor skills is also associated to light sleep, and the brain processes and consolidates information by transferring information from short-term to long-term memory (Nishida and Walker, 2007). The light sleep stage also helps to restore the brain’s cognitive processes. A football game is both physical-and cognitive demanding, and an increase in light sleep might therefore improve recovery, especially when both deep-and REM-sleep increase at the same time.

An increase in REM sleep after football games will help the players to regulate their emotions, consolidate experienced episodes in the game related to the play–counter play interactions, and recover from muscular damage caused by physical efforts in the game (Vgontzas et al., 1997; Peever and Fuller, 2017). The hypothalamic–pituitary–adrenal (HPA) axis is found to be activated in REM sleep and modulates the stress response system by stimulating the adrenal release of cortisol (Chrousos et al., 2016). Football games at elite level often result in potential muscle fiber micro ruptures which lead to inflammatory processes (Nédélec et al., 2013), and cortisol is found to reduce such inflammations (Braun and Marks, 2015). Interestingly, a recent study among female football players at international level, shows that increases in REM sleep were associated with subsequently decreased perceived fatigue the next day (Moen et al., 2021). Thus, loads associated with football games seem to increase the need for higher amounts of REM sleep to be fully recovered.

An increase in deep sleep after football games will help the players to obtain physical restoration, since deep sleep helps our bodies to repair, regenerate and restore from the extraordinary loads associated with football games (Nédélec et al., 2015). The endocrine system increases the secretion of growth hormones in deep sleep, which allows the muscles to regenerate and grow (Åkerstedt and Nilsson, 2003; Halson, 2014). Other hormones also increase during deep sleep, such as hormones that regulates the stress response and melatonin, which helps to reduce stress and promote relaxation. Therefore, deep sleep has an especially vital function for physical recovery (Doherty et al., 2021). Deep sleep also regulates learning, where information from the day (e.g., from the game) are transferred from short-term to long-term memory, which is important in football that is a dynamic sport where decisions must be made in the moment based on information that is recalled from episodic memory. Therefore, deep sleep is needed for football players to be fully recovered from games (Knufinke et al., 2018), and that the combination of REM-sleep and deep sleep seem essential in the recovery process after extraordinary loads.

However, the results also show that sleep onset latency is higher in the experiment period on the night after the night after game (game night +1), and that sleep efficiency is lower on game night, and that wake after sleep onset is higher on all nights related to game night in the experiment period compared to the control period (Table 5 and Figure 3). Taken together, the results are a bit contradictive to claim that sleep quality is improved after the introduction of BEMER therapy. On the one side, sleep variables that are used to consider sleep quality such as total sleep time, light sleep, deep sleep, and REM sleep have increased after the introduction of BEMER therapy. On the other side, sleep variables such as sleep onset latency and wake after sleep onset have increased, which is negative, and sleep efficiency is reduced on game night. However, when BEMER time is included in the analyses as a control, the results show that sleep onset latency is significantly lower, and that total sleep time, sleep efficiency, light sleep, and REM sleep are higher in the group of players who spent BEMER therapy more than 440 h in the experimental period (Figure 4). The results also show that deep sleep is significantly increased among the players who spent more than 440 h compared to the group that spent BEMER therapy less, and that the group with less use had an increase in wakening’s after sleep onset. The results further show that that in the group of players that spent BEMER therapy more than 440 h, sleep onset latency was significantly reduced on game nights in the experiment period, and that total sleep time, sleep efficiency, light sleep, REM sleep and deep sleep are significantly increased in the experiment period on game nights and nights associated to game nights (Figure 5). It is also worth noting that all sleep variables were reduced significantly below the players’ baselines on ordinary training days on game night in the control period, whereas the sleep variables are close to or above their baselines on game nights in the experimental period (Figures 3, 5). Thus, in total, the results in the current study indicate that sleep quality is significantly improved after the introduction of BEMER therapy.

### 4.3. Conclusion, limitations, and strengths

Earlier studies claim that elite football players obtain suboptimal recovery on game nights because of the extraordinary loads caused by the game (Moen et al., 2021), and that sleep loss have a negative impact on exercise performance (Craven et al., 2022). The current study also shows that the players’ total sleep time and their distributed time in light-, deep-and REM-sleep drop on game night, while the objective measured loads peak on game night. Interestingly, the players obtain significantly more total sleep time and distributed time in light-, deep-, and REM-sleep after the introduction of BEMER therapy. BEMER therapy therefore seems to be an efficient recovery tool since the state of sleep represents the best natural enhancer for recovery (Bonnar et al., 2018). However, some limitations should be kept in mind when interpreting the current results. First, what sleep need football players have after completing extraordinary loads on game nights, and what distribution between the different sleep stages that are optimal, are not fully understood. The results should therefore be interpreted with caution. Second, the study would have benefitted from a larger sample size and a control group. The relatively low number of participants may have influenced the power of the conducted statistical analyses and is therefore a limitation in the current study. Third, the loss of all potential sleep data may also have influenced the results. Fourth, the players might have been influenced by seasonal effects such as different stress levels throughout the pre-season and season, to different training-related physiological levels throughout the pre-season and season, and to seasonal differences in hours of daylight. The current study has several strengths as well. First, the participants in the current study are female football players in a Norwegian top series club. Second, the data collection lasted for 9 months, which resulted in many data points for each player. Third, the intervention was designed with a control period for each player, and the analyses controlled for the actual time players used BEMER therapy. The analyses and results that included BEMER therapy time strengthen the design in the current study.

## Data availability statement

The raw data supporting the conclusions of this article will be made available by the authors, without undue reservation.

## Ethics statement

The studies involving humans were approved by Research Ethics (REC) in Central Norway (project ID 2017/2072/REK midt). The studies were conducted in accordance with the local legislation and institutional requirements. The participants provided their written informed consent to participate in this study.

## Author contributions

FM, SAP, and AK contributed to the conception and design of the study. EM, FM, and KL performed the statistical analysis. FM wrote the first draft of the manuscript. FM, MV, KG, AK, and MR organized the logistics during the experiment, and FM, MV, KG, MR, and SAP organized the database. EM wrote sections of the manuscript. FM and SAP contributed to the main revision of the manuscript. FM, SAP, and EM contributed to the final manuscript revision. All authors contributed to the article and approved the submitted version.

## Funding

This study was funded by the Department of Education and Lifelong Learning, Norwegian University of Science and Technology, Trondheim, Norway, Tromsø Research Foundation (grant number 19_FFC_SAP), and Precision Food Production (grant number 314111) at Nofima AS - The Norwegian Institute of Food, Fisheries and Aquaculture Research, and Faculty of Science and Technology at Norwegian University of Life Science.

## Conflict of interest

The authors declare that the research was conducted in the absence of any commercial or financial relationships that could be construed as a potential conflict of interest.

## Publisher’s note

All claims expressed in this article are solely those of the authors and do not necessarily represent those of their affiliated organizations, or those of the publisher, the editors and the reviewers. Any product that may be evaluated in this article, or claim that may be made by its manufacturer, is not guaranteed or endorsed by the publisher.

## Footnotes

^1^ https://cran.r-project.org/package=ffmanova
